# Air Quality Before and After COVID-19 Lockdown Phases Around New Delhi, India

**DOI:** 10.5696/2156-9614-11.30.210602

**Published:** 2021-05-28

**Authors:** Sudesh Chaudhary, Sushil Kumar, Rimpi Antil, Sudesh Yadav

**Affiliations:** 1 Centre of Excellence for Energy and Environmental Studies, Deenbandhu Chhotu Ram University of Science and Technology, Murthal, India; 2 School of Environmental Sciences, Jawaharlal Nehru University, New Delhi, India

**Keywords:** air pollution, lockdown, meteorology, criteria pollutants

## Abstract

**Background.:**

The COVID-19 pandemic has had a deep global impact, not only from a social and economic perspective, but also with regard to human health and the environment. To restrict transmission of the virus, the Indian government enforced a complete nationwide lockdown except for essential services and supplies in phases from 25 March to 31 May 2020. Ambient air quality in and around New Delhi, one of the most polluted cities of world, was also impacted during this period.

**Objective.:**

The aim of the present study was to assess and understand the impact of four different lockdown phases (LD1, LD2, LD3 and LD4) on five air pollutants (particulate matter (PM) PM_2.5_, PM_10_, nitrogen oxide (NO_x_), sulfur dioxide (SO_2_) and ozone (O_3_)) compared to before lockdown (BLD) at 13 air monitoring stations in and around New Delhi.

**Methods:**

Secondary data on five criteria pollutants for 13 monitoring stations in and around New Delhi for the period 1 March to 31 May 2020 was accessed from the Central Pollution Control Bard, New Delhi. Data were statistically analyzed across lockdown phases, meteorological variables, and prevailing air sources around the monitoring stations.

**Results.:**

Pollutant concentrations decreased during LD1 compared to BLD except for O_3_ at all stations. PM_2.5_ and PM_10_ remained either close to or higher than the National Ambient Air Quality Standards (NAAQS) due to prevailing high-speed winds. During lockdown phases, NO_2_ decreased, whereas O_3_ consistently increased at all stations. This was a paradoxical situation as O_3_ is formed via photochemical reactions among NO_x_ and volatile organic compounds. Principal component analysis (PCA) extracted two principal components (PC1 and PC2) which explained up to 80% of cumulative variance in data. PM_2.5_, PM_10_ and NO_2_ were associated with PC1, whereas PC2 had loadings of either O_3_ only or O_3_ and SO_2_ depending upon monitoring station.

**Conclusions.:**

The present study found that air pollutants decreased during lockdown phases, but these decreases were specific to the site(s) and pollutant(s). The decrease in pollutant concentrations during lockdown could not be attributed completely to lockdown conditions as the planetary boundary layer increased two-fold during lockdown compared to the BLD phase. Such restrictions could be applied in the future to control air pollution but should be approached with caution.

**Competing Interests.:**

The authors declare no competing financial interests.

## Introduction

Economies across the world came to halt after the World Health Organization (WHO) released the news of the outbreak of a new virus, SARS-CoV-2 (coronavirus) on 5 January 2020 and later declared a global pandemic on 12 March 2020.^1^,^2^ In India, the number of infections has crossed the 9.8 million mark with 140 000 deaths through 12 December 2020 since the first case of COVID-19 in India was reported on 30 January 2020 in the state of Kerala.^3^ Like other countries, India adopted lockdown strategies to restrict the spread of the virus. Public movement and transport (road/rail/air) were restricted, and industrial operations, governmental, as well as private institutions, offices, and shopping complexes were shut. Only essential services (medical, banking, daily consumables, media, communications etc.) remained in operation during the lockdown phases. Air pollution scientists considered this unexpected and sudden lockdown to be a blessing in disguise for combating air pollution.^4^,^5^,^6^,^7^,^8^,^9^ Wuhan city in China observed a nearly 63% and 35% reduction in nitrogen dioxide (NO_2_) and particulate matter (PM) concentrations in the ambient atmosphere, respectively, with limited effects on sulfur dioxide (SO_2_) and carbon monoxide (CO) after lockdown began on 23 January 2020.^10^ Similarly, the town of Southampton in the United Kingdom observed a 92% reduction in NO_2_ emissions due to lockdown on 23 March 2020 compared to the same period from 2017–19.^10^ In general, studies emerging from different countries have reflected a reduction in air pollution during lockdown periods.

In India, a complete lockdown, termed the Janta Curfew, was imposed on 22 March 2020 followed by complete lockdown phase 1 (LD1) (25 March–14 April 2020) with an almost complete ban on all activities except for essential supplies. In LD2 (15 April–3 May 2020), selected agricultural and industrial activities were allowed, and in LD3 (4–17 May 2020) and LD4 (18–31 May 2020), industrial and construction activities and other limited operations were allowed. These measures had a significant impact on criteria pollutants in ambient atmosphere.^5^,^11^ Previous reports observed a nearly 44% reduction of PM_10_, 8% of PM_2.5_, 44% of nitrogen oxide (NO_X_)_,_ and 32% of CO in New Delhi during the Janta curfew.^12^ This sharp decline in pollution levels was due to restrictions on transport services, construction work and industrial activities. Air quality index (AQI) values varied between satisfactory to moderate during the lockdown phases in the National Capital Region (NCR) of New Delhi.^12^ Overall, decreases of 43%, 31%, 18%, and 10% in PM_2.5_, PM_10_, NO_2,_ and CO levels, respectively, were reported during lockdown in India, whereas ozone (O_3_) showed a 17% increase in concentration and SO_2_ showed site-specific variations.^5^ Kolkata, in eastern India, experienced a reduction of 24% to 45% in emissions of CO during lockdown due to limited industrial emissions and transport services.^13^ Similarly, AQI at Silicon Valley (Bengaluru) in southern India improved from the ‘hazardous' category after lockdown.^14^ A nearly 40–50% reduction in NO_2_ levels were reported in New Delhi and Mumbai during lockdown.^4^,^15^ During lockdown, particulate matter concentrations were at the lowest recorded levels in the previous 20 years.^4^,^15^ A significant reduction in air pollutants was observed during lockdown compared to 2017 and 2019 data over Kanpur, the Indo-Gangetic Plain and northern India.^16^,^17^ The lockdown provided an opportunity to observe and study the impacts of reduced emissions from different sources on ambient air quality. Such studies can help us to control air pollution in the future. New Delhi and its surrounding regions are among the most polluted parts of India and therefore, the present study aimed to observe the response of criteria pollutants during lockdown phases compared with the period immediately prior as well as with the same period in previous years.

**Abbreviations**
*BLD*: Before lockdown
*CPBC*: Central Pollution Board Control
*IGIA-T3*: Indira Gandhi International Airport-Terminal 3
*LD*: Lockdown
*NAAQS*: National Ambient Air Quality Standards
*PBL*: Planetary boundary layer
*PCA*: Principal component analysis
*VOC*: Volatile organic compounds
*WHO*: World Health Organization

In the present study, the responses of five criteria pollutants: PM_1_0, PM_2.5_, SO_2_, NO_x,_ and O_3_were observed and analyzed for the period before lockdown (BLD: 1–24 March 2020), and the different lockdown phases: LD 1 (25 March–14 April 2020), LD2 (15 April–3 May 2020), LD 3 (4–17 May 2020) and LD 4 (18–31 May 2020). A comparison of air quality data for March to May 2020 was made with air quality data during the same period in 2019.

## Methods

The present study was carried out using secondary data collected from the Central Pollution Control Board (CPCB) for five criteria pollutants during the period before lockdown (BLD) and during different phases of lockdown in and around New Delhi. Meteorological data (temperature, relative humidity, planetary boundary layer and wind speed) were used to understand their possible role in variations in mass concentrations of pollutants. Furthermore, principal component analysis (PCA) was used to extract the number of principal components based on inter-pollutant associations and each component was assigned as a possible source. Principal component analysis was carried out on all five pollutants for all thirteen monitoring stations using the Statistical Package for the Social Sciences (SPSS) software version 20 using the varimax rotation method. Only components with an eigen value greater than unity were considered following the Kaiser criteria.^18^,^19^

### Study area and data collection

**Figure 1:**
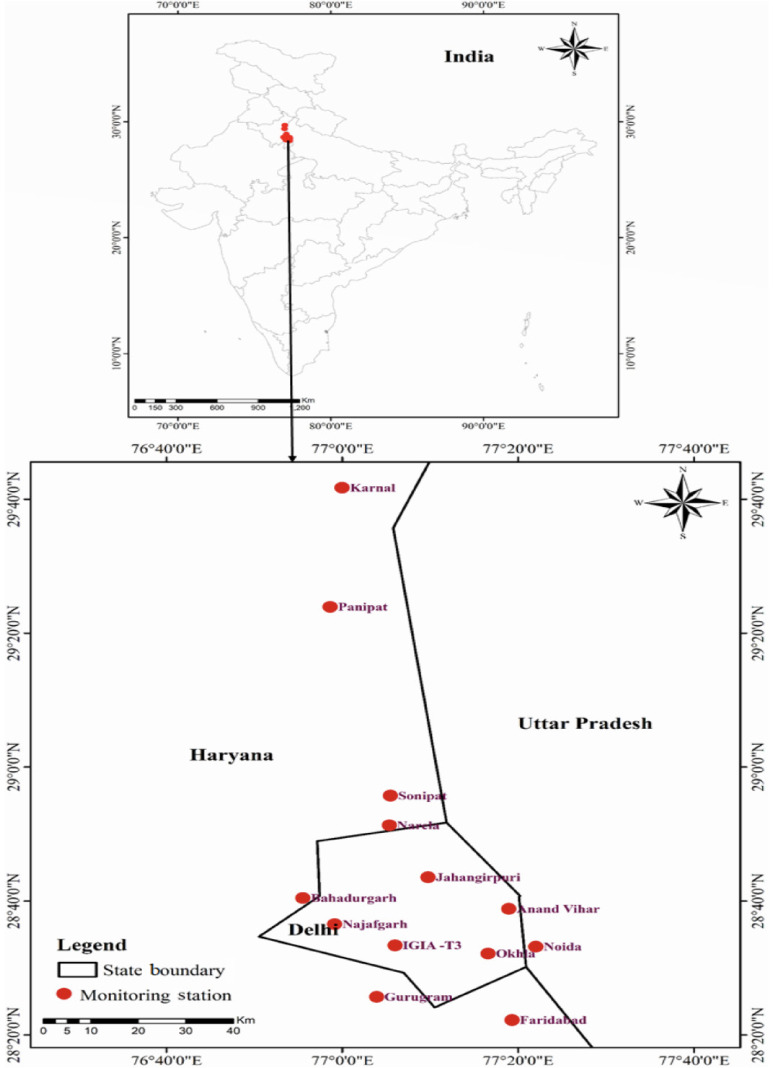
Map showing the geographical coordinates of the selected monitoring stations used for data collection in the present study. All stations are installed and maintained by the Environment Information System (ENVIS) Resource Partner on Control of Water, Air and Noise Pollution hosted by the Central Pollution Control Board (CPCB) and sponsored by the Ministry of Environment, Forests and Climate Change, Government of India.^21^

Air quality data for five criteria pollutants were taken for total six monitoring stations (Anand Vihar, Narela, Indira Gandhi International Airport-Terminal-3 (IGIA-T3), Najafgarh, Okhla and Jahangirpuri) covering New Delhi, three stations (Sonipat, Panipat and Karnal) in the state of Haryana along the national highway emerging from New Delhi from the north, and Bahadurgarh, Gurugram, Faridabad and Noida, located along other national highways emerging out of New Delhi *(Figure 1).* Sonipat, Bahadurgarh, Gurugram, Faridabad and Noida share their boundaries with New Delhi. These selected monitoring stations are located in areas that are a mix of urban residential, airport, industrial (Bahadurgarh), construction (Noida and Gurugram), agricultural fields (Sonipat, Panipat and Karnal), etc. All monitoring stations in the present study are located in areas of high air pollution due to vehicular and industrial emissions. The climate in this region during the study period (March–May 2020) remained semi-arid with hot and dry conditions and intermittent dust storms (Aandhi) originating from the Great Indian Thar desert in western India.^18^,^19^,^20^

The 24-hr daily average data of criteria pollutants, i.e., particulate matter ≤ 2.5 μm (PM_2.5_), particulate matter ≤ 10 μm (PM_10_), NO_2_,SO_2_ and O_3_ used in this study were taken from the Environment Information System (ENVIS) resource partner on Control of Water, Air and Noise Pollution hosted by the CPCB and sponsored by the Ministry of Environment, Forests and Climate Change (MoEFCC) of the government of India.^21^ These data are released in the public domain by the CPCB after quality control and corrections, if required. The data were divided into the phases of BLD, and LD1, LD2, LD3 and LD4 and the minimum and maximum concentrations of pollutants and their standard deviations were calculated for these respective phases. Daily meteorological data were collected from the Air Resource Laboratory- National Oceanic Atmospheric Administration (ARL-NOAA).^14^ Average temperature and humidity over all the monitoring stations ranged between 19.4 to 21.7ºC and 46.7 to 60.8%, respectively, in the period before lockdown *(Table 1).* Similarly, average temperature and humidity ranged from 28.4 to 31.9ºC and 23.8 to 30.1% over all the monitoring stations during the lockdown phases. The planetary boundary layer (PBL) and wind speed showed significant variations before lockdown and during lockdown phases. Average PBL and wind speed ranged between 426.6 to 593.3 m and 8.0 km hr^−1^ to 11.4 km hr^−1^ during before lockdown, whereas average PBL and wind speed varied between 939.9 m to 1226.0 m and 8.6 km hr^−1^ to 13.8 km hr^−1^ at all monitoring stations during lockdown phases (*Table 1*). A significant increase in the PBL is expected to have a pronounced effect on pollutant concentrations in ambient atmosphere. Increase in PBL height leads to a decrease in concentrations due to dispersion of pollutants in larger volume in ambient atmosphere. Variations in concentrations of pollutants are discussed in conjunction with PBL and wind speed data.

**Table 1:** Meteorological data over selected monitoring station around New Delhi before Lockdown (BLD: 1 to 24 March 2020) and During Lockdown (LD: 25 March to 31 May 2020)

	**Period**	**Temp (°C)**	**RH (%)**	**PBL (m)**	**WS (km/hr)**
Anand Vihar	BLD	20.4±5.2	53.1±19.0	457.9±740.3	8.0±5.1
	LD	28.4±5.9	30.1±14.4	939.9±1255.4	10.3±5.2
Narela	BLD	19.4±4.7	58.4±20.3	528.5±710.8	11.4±6.6
	LD	30.4±5.8	29.9±15.7	1118.4±1346.0	11.4±5.5
IGIA-T3	BLD	20.0±5.0	52.3±19.4	533.5±763.1	9.3±5.3
	LD	30.6±5.7	28.9±14.9	1128.5±1353.8	10.8±5.3
Najafgarh	BLD	19.6±4.8	55.0±20.6	542.2±750.7	10.3±6.0
	LD	30.7±5.7	29.1±14.9	1128.4±1355.3	10.9±5.3
Okhala	BLD	19.8±4.7	55.4±20.1	538.9±741.2	9.7±5.7
	LD	30.6±5.7	28.9±14.9	1125.2±1343.1	10.7±5.2
Sonipat	BLD	19.0±4.8	60.8±20.7	498.6±690.4	11.8±7.1
	LD	30.8±5.7	29.8±16.3	1157.3±1380.0	11.8±5.8
Panipat	BLD	19.7±6.4	49.5±18.1	426.4±547.5	8.9±4.7
	LD	31.6±6.3	24.3±11.9	1151.9±1459.0	8.9±4.4
Jahagirpuri	BLD	20.2±6.5	46.7±17.1	463.3±592.4	8.3±4.6
	LD	31.8±6.2	23.8±11.3	1179.2±1464.1	8.6±4.3
Karnal	BLD	19.7±5.2	59.8±19.0	479.4±646.2	10.1±6.1
	LD	30.6±6.3	29.5±16.1	1157.5±1386.2	12.1±5.9
Gurugram	BLD	21.7±5.3	48.2±19.2	593.3±857.6	8.9±5.3
	LD	31.9±5.9	26.2±14.1	1219.3±1459.0	11.1±5.7
Bahadurgarh	BLD	20.6±5.8	49.2±18.2	590.3±858.8	8.6±4.3
	LD	30.6±4.6	24.1±14.1	1213.8±1452.0	13.8±5.2
Faridabad	BLD	21.7±5.3	48.9±19.3	588.1±849.8	8.8±5.2
	LD	31.9±5.9	25.9±14.1	1226.0±1457.8	10.9±5.6
Noida	BLD	21.5±5.3	50.7±19.4	574.6±821.2	8.9±5.4
	LD	31.7±6.0	26.5±14.4	1215.6±1445.0	11.01±5.7

Abbreviations: PBL(m), Planetary boundary layer in meters (m); RH, Relative humidity; WS (km/hr), Wind speed in km/hr.

## Results

Average concentrations of PM_2.5_, PM_10_, NO_2_, SO_2_ and O_3_ with their range (minimum and maximum) and standard deviation for 13 monitoring stations during before lockdown (BLD) and lockdown phases LD1, LD2, LD3, and LD4 are given in Supplemental Material Table 1 and are shown graphically in Supplemental Material Figure 1. The percentage increase or decrease in pollutant concentrations were calculated with respect to their concentrations in the preceding phase and are given in Supplemental Material Table 1. Average metrological data for all 13 monitoring stations for the BLD period (1 to 24 March 2020) and during lockdown phase from 25 March to 31 May 2020 are provided in Table 1. Moving from BLD to the lockdown phases, ambient temperature increased, and relative humidity decreased by half at almost all of the stations. Wind speed either remained similar at some stations or increased at other stations *(Table 1).* An important meteorological feature that changed was the PBL, which was increased by more than two-fold across all stations during the lockdown phase compared to BLD.

The concentrations of PM_2.5_ and PM_10_ ranged from 19.6 to 176.5 μg m^−3^and 42.5 μg m^−3^ to 325.8 μg m^−3^, respectively, over New Delhi with the highest average concentration observed at Jahagirpuri (104.5μg m^−3^ and 224.3 μg m^−3^) followed by Anand Vihar (78.1 μg m^−3^ and 193.8 μg m^−3^), Narela (71.9 μg m^−3^and 165.9 μg m^−3^), Najafgarh (69.3 μg m^−3^ and 133.7 μg m^−3^), Okhla (61.5 μg m^−3^ and 146.8 μg m^−3^) and IGIA-T3 (55.3 μg m^−3^ and 136.3 μg m^−3^) during the BLD phase. Average concentrations of PM_2.5_ and PM_10_, respectively, over monitoring stations located along the northern national highway (Sonipat: 33.2 μg m^−3^and 132.9 μg m^−3^; Panipat: 38.7 μg m^−3^ and 94.8 μg m^−3^; Karnal: 51.2 μg m^−3^ and 100.5 μg m^−3^) were lower than the National Ambient Air Quality Standards (NAAQS) except at Sonipat for PM_10_. The other monitoring stations around New Delhi showed high average concentrations of PM_2.5_ and PM_10_, respectively, (Bahadurgarh: 61.6 μg m^−3^and 101.2 μg m^−3^; Gurugram: 71.3 μg m^−3^ and 154.5 μg m^−3^; Faridabad: 71.9 μg m^−3^and 174.5 μg m^−3^) and exceeded the NAAQS except at Noida (57.4 μg m^−3^ and 136.7 μg m^−3^).

On implementation of LD1, average concentrations of PM_2.5_ and PM_10_ showed a significant reduction compared to the BLD phase over New Delhi and surrounding stations except for some monitoring stations (*Supplemental Material Table 1 and Supplemental Material Figure 1*). Average reductions in PM_2.5_ and PM_10_ during LD1 compared to BLD over New Delhi varied between 20 to 60% (Anand Vihar: −57 % and −59.2%; Narela: −40.8% and −35.6%; IGIA-T3: −37.4% and −1.%; Najfgarh: −26.9% and +10.0%; Okhla; 38.2% and −45.9%) except at Jahagirpuri station (+17.2% and +20.5%) (*Supplemental Material Table 1 and Supplemental Material Figure 1*). A reduction in average concentrations of PM_2.5_ and PM_10_ was observed over Sonipat (−31.6 % and −36.1%) and Karnal (−36.5 % and −35.4 %), whereas Panipat station, located between Sonipat and Karnal, showed an increase of 0.28% and 62.2%, respectively. Average concentrations of PM_2.5_ and PM_10_ at other stations also showed significant reductions, respectively (Bahadurgarh: −40.4% and −34.1%; Gurugram: −46.1% and −45.5%; Faridabad: −55.6% and −51.2%; Noida: −41.4% and −36.5%) compared to the BLD phase on account of lockdown. On implementation of LD2, PM_2.5_ and PM_10_ concentrations increased slightly over New Delhi compared to LD1 due to gradual lifting of restrictions from certain activities.The highest increases in PM_2.5_ and PM_10_ concentrations were observed at Anand Vihar (+52.9% and +38.8%) followed by Narela (+41.5% and +46.5%), Okhla (+13.6% and 35.8%), and IGIA-T3 (+11.8% and +25.7%). However, Najafgarh (−16.9% and −3.2%) and Jahagirpuri (−21.2% −3.2%) again showed a decrease in concentrations of particles compared to the LD1 period. Monitoring stations located along the national highway showed increased concentrations compared to LD1. Sonipat showed the highest increase in PM_2.5_ and PM_10_ (+54.1% and +75.8% ) followed by Karnal (+60.9% and +27.4%) and Panipat (+0.28% and +62.2%). Similarly, other monitoring stations around New Delhi showed an increase in PM_2.5_ and PM_10_ concentrations in LD2 compared to LD1. Average concentrations of PM_2.5_ and PM_10_ further increased in LD3 and LD4 compared to LD2 and LD3, respectively. Compared to the same period in the previous year (2019), PM_2.5_ and PM_10_ concentrations in the BLD phase were nearly the same at all stations except that concentrations were higher at Panipat *(Supplemental Material Figure 2).* Similarly, PM_2.5_ and PM_10_ concentrations during LD1 to LD4 were lower at all stations compared to the corresponding period in the previous year (2019) (*Supplemental Material Figure 2*).

The average concentration of NO_2_ was highest over the New Delhi stations (Anand Vihar: 54.5 μg m^−3^; Jahagirpuri: 50.6 μg m^−3^; IGIA-T3: 48.2 μg m^−3^; Narela: 39.6 μg m^−3^; Okhla: 36.3 μg m^−3^ and Najafgarh: 26.5 μg m^−3^) compared to other monitoring stations and below the NAAQS during the BLD phase (*Supplemental Material Table 1 and Supplemental Material Figure 1*). In the present study, NO_2_ concentrations over New Delhi exceeded the WHO standards except at Anand Vihar, Jahagirpuri, and IGIA-T3. In addition, NO_2_ concentrations along the national highway stations (Sonipat: 46.8 μg m^−3^; Panipat: 47.6 μg m^−3^ and Karnal: 17.4 μg m^−3^) remained below the NAAQS. During LD1, NO_2_ showed significant reductions over New Delhi followed by other stations along the national highways. The highest reduction in NO_2_ concentration was observed at IGIA-T3 (−83.6%) followed by Anand Vihar (−65.6%), Okhla (−63.0%), Najafgarh (−57.3%) compared to the BLD period. However, the Jahagirpuri station showed an increase in NO_2_ concentrations compared to BLD. Monitoring stations along the national highways also showed significant reductions in NO_2_ concentration (Karnal:−64.9%; Panipat: −5.9%; Noida: −208.6%; Gurugram: −39.6% and Bhadurgarh: −28.8%) except Sonipat (+19.2%) and Faridabad (+25.3%). During successive implementation of LD2, LD3 and LD4, NO_2_ concentrations showed monitoring station-specific as well as lockdown phase-specific decreases or increases (*Supplemental Material Table 1 and Supplemental Material Figure 1*). Compared to previous year observations during the same period, NO_2_ concentrations were lower at all stations except for Sonipat and Panipat along the national highway (*Supplemental Material Figure 2*).

The average concentration of O_3_ was highest over New Delhi (Narela: 47.8 μg m^−3^; Najafgarh: 46.7 μg m^−3^; Jahangirpuri: 37.7 μg m^−3^; Anand Vihar: 32.8 μg m^−3^; Okhla: 29.8 μg m^−3^; and IGIA-T3:12.5 μg m^−3^) followed by other stations along national highways (Panipat: 27.9 μg m^−3^; Sonipat: 20.2 μg m^−3^; Karnal: 18.9 μg m^−3^; Gurugram: 59.0 μg m^−3^; Bahadurgarh: 34.5 μg m^−3^; Noida: 29.3 μg m^−3^; and Faridabad: 26.6 μg m^−3^) during BLD. Average O_3_ concentrations were below the limits of NAAQS and WHO guidelines. The O_3_ concentration also remained lower than standard limits during the lockdown phases. However, the O_3_ concentration consistently increased upon relaxation of lockdown phases except at Najafgarh and Noida stations (*Supplemental Material Table 1 and Supplemental Material Figure 1*). Unlike NO_2_, O_3_ increased over New Delhi (Anand Vihar: +81.7%; IGIA-T3: +79.2%; Okhla: +46.6%; Jahangirpuri: +42.3%; Najafgarh: +5.1%) except at Narela (−2.7%) and all other monitoring stations (Karnal: +34.9%; Panipat: +25.3%; Bahadurgarh: +21.4%, and Gurugram: +18.8%; except Sonipat: −27.9%; Noida: −37.5%; and Faridabad: −12.8%) during LD1 (*Supplemental Material Table 1*). In addition, O_3_ concentrations further increased in successive phases of lockdown (LD2, LD3 and LD4). Compared to observations during the same period in 2019, average O_3_ concentrations showed a mixed trend over New Delhi with higher O_3_ concentrations at Anand Vihar and Jahangirpuri in all phases. Furthermore, the monitoring stations (Narela, IGIA-T3, Najafgarh, Okhla) showed lockdown phase-specific trends (increase/decrease) in O_3_ concentrations compared to the same period in the year 2019 (*Supplemental Material Figure 2*). Monitoring stations along the national highway (Sonipat and Karnal) showed lower O_3_ concentrations compared to the previous year, except for Panipat. However, Bahadurgarh and Noida showed higher O_3_ concentrations in all phases (except LD4 at Noida) compared to the previous year.

The SO_2_ concentrations at all stations remained well within the NAAQS limits, but exceeded WHO limits. Concentrations of SO_2_ over New Delhi stations and Sonipat and Panipat remained similar throughout the study period except for a decline at IGIA-T3 station (*Supplemental Material Table 1 and Supplemental Material Figure 1*). Other stations at Bahadurgarh, Gurugram and Faridabad showed a consistent decrease in SO_2_ concentration, whereas the Noida station showed an increase from BLD to LD4. Overall, SO_2_ concentrations were not generally affected except at a few stations where they showed a decline during the LD period. Compared to the same time period in the previous year (2019), SO_2_ concentrations showed a decreasing trend at all stations due to lockdown (*Supplemental Material Figure 2*). Monitoring stations located along the national highway (Sonipat and Panipat) except for Karnal showed either equal or lower concentrations compared to the same time period in the previous year, 2019. The SO_2_ concentration at Bahadurgarh remained high in all phases except for Noida compared to the previous year's data (*Supplemental Material Figure 2*).

Output data of PCA on the number of components with their eigen values and percentage variance explained by each component as well as cumulative variance explained by all components are presented in Supplemental Material Table 2. There were only two PCs across all monitoring stations except for Karnal and Noida wherein only one component was extracted. In general, PC1 explained the data variance in the range of 41 to 62%, whereas PC2 could explain 20 to 34% of the variance in data across all stations. In addition to loadings of PM_2.5_ and PM_10_ in PC1 at all stations, loadings of NO_2_ and SO_2_ and were also observed at some stations. The PC2 has always carried loadings of O_3_ with addition of SO_2_ at some stations.

## Discussion

In general, the present study observed a decrease in concentrations of all pollutants across all monitoring stations during the LD1 phase compared to BLD. In LD2 and LD3, the concentrations either remained the same or increased/decreased depending on pollutant(s) and location(s).

The PM_2.5_ and PM_10_ concentrations exceeded NAAQS limits over New Delhi (Anand Vihar, Narela, IGIA-T3, Najafgarh, Okhla and Jahagirpuri) and surrounding monitoring stations (Bahadurgarh, Gurugram, Faridabad and Noida) except for Sonipat, Panipat and Karnal during BLD. Average reduction in PM_2.5_ and PM_10_ concentrations over New Delhi varied from 20 to 60% during LD1 compared to BLD due to sudden restrictions on public movement, industrial activity, and public transport (*Supplemental Material Table 1 and Supplemental Material Figure 1*). High PM_2.5_ concentrations over New Delhi could be associated with traffic emissions and biomass burning activities, while PM_10_ concentrations were largely associated with resuspension of road dust, local emissions, and construction activity.^20^ Lower concentrations at monitoring stations (Sonipat and Karnal) along the northern national highway were due to restrictions on emission sources and precautions taken by the general public to restrict unnecessary movement before lockdown. Increases in particle concentrations at Panipat during LD1 could be attributed to local factors such as ploughing in fields and industrial actvities. High concentrations of PM_2.5_ and PM_10_ at other stations (Bahadurgarh, Gurugram, Faridabad, Noida) aound New Delhi could be due to construction activities around these stations and wind blown dust or local resuspension of particles as has also been reported previously for this region.^18^,^19^,^20^ PM_2.5_ and PM_10_ concentrations increased slightly during LD2 after LD1 due to gradual lifting of restrictions of certain activities. In addition, the duration of LD2 coincided with onset of strong south westerly winds which could bring intermitent dust storms and amplify concentrations of PM_2.5_ and PM_10_.^18^,^19^,^20^ Further increases in PM_2.5_ and PM_10_ concentrations during LD3 and LD4 could be attributed to further relaxation on public and vehicular movement, resuspension of surface dust, and biomass burning in and around the monitoring stations. Similar to our observations, a nearly 89% decline in PM_10_ concentrations was observed at the Central Road Research Institute, New Delhi station due to lockdown.^22^ At North Campus, Delhi University, New Delhi, PM_2.5_ concentrations decreased from 225.63 μg m^−3^ before lockdown to 43.18 μg m^−3^ after lockdown.^23^ Jain and Sharma reported significant reductions of 41% and 52% in the concentrations of PM_2.5_ and PM_10_, respectively, due to lockdown in New Delhi.^24^ A decline of 45% was observed in PM_2.5_ concentration in Wuhan city, China due to the pandemic.^2^ The decrease in PM_2.5_ and PM_10_ concentrations at all stations due to lockdown clearly indicated that lockdown acted as a ventilator for the ambient atmosphere, although PM_10_ and PM_2.5_ were not within NAAQS and WHO limits even after lockdown in the study region except for PM_2.5_ at Sonipat during LD1. High concentrations of PM_2.5_ and PM_10_ compared to WHO and NAAQS limits over north India could be a geographical location-linked phenomenon. The time period in the present study overlapped with the onset of intermittent dust storm events due to strong prevailing winds. Compared to the same period in 2019, PM_2.5_ and PM_10_ showed a decline, again due to restrictions (*Supplemental Material Figure 2*). A slight increase in PM concentrations was noticed at the Panipat site, which can be attributed to local factors like coal energy-based thermal power plants which were operational during the lockdown period and re-suspension of emissions.

Vehicular emissions are a major source of NOx (NO and NO_2_) along with power plants and industries. A secondary criteria air pollutant, O_3_ is formed via photochemical reactions among NOx and volatile organic compounds (VOCs). The formation of O_3_ in ambient atmosphere is facilitated during the summer season due to the presence of sufficient sunlight for photochemical reaction as given below in Equation 1.^25^,^26^


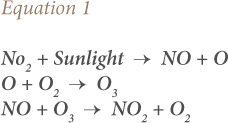


The concentration of NO_2_ continued decreasing at all monitoring stations during LD 1 compared to BLD due to restrictions on vehicular movement except for essential services. Similarly, a 50% decrease in NO_2_ concentration was observed at New Delhi due to lockdown.^24^ The thermal power plant, located near Fridabad which was operational during lockdown, could be the possible reason for this decrease, whereas small scale industries of essential items and agricultural fields around Sonipat could explain the increase in NO_2_. Similarly, the increase in NO_2_ during LD3 and LD4 could be due to greater relaxation of restrictions and restarting of industrial operations.

Due to restrictions on vehicular movement and limited NO_2_ emissions, O_3_ remained lower than prescribed standards at all stations during lockdown phases. The increase in O_3_ concentrations over New Delhi except for in Narela during LD1 could be related to the availability of NO_x_ and VOCs. The monitoring stations along national highways (Karnal and Bahadurgarh) could have been influenced by NOx emissions from wetlands or crop residue burnings which could have led to more O_3_ formation during lockdown phases. Sources of NO_2_ in Panipat included vehicles, oil refineries, thermal power plants, and fertilizer industries.^27^,^28^ Similar to Panipat and Jahangirpuri, an increase of 7% in O_3_ concentrations in New Delhi and 3% in Chennai has been observed.^24^ Xu *et al.* also reported an increase in O_3_ concentrations in Wuhan city in China during the lockdown period.^29^ The increase in O_3_ concentrations in 2020 compared to the same time period in the previous year could be due to decreased concentrations of particulate matter which resulted in greater penetration of sunlight in the atmosphere, resulting in an increase in photochemical activity and hence greater O_3_ formation.^5^

Industries and coal-based thermal power plants are responsible for nearly 27% of SO_2_ concentration in and around New Delhi.^27^ However, SO_2_ concentrations at all stations remained well within the NAAQS limits. Slight changes in SO_2_ concentrations at Panipat and Karnal stations could be due to emissions from the thermal power plant, fertilizer company and oil refineries operating around the city during LD2 and LD4.^30^,^31^ The lowest concentration of SO_2_ at the IGIA-T3 was a visible effect of lockdown. Decreased SO_2_ concentrations around Bahadurgarh, Gurugram and Faridabad stations could be linked to migration of the labor class, which use coal as cooking fuel. The restarting of activities could have increased SO_2_ concentrations from BLD to LD4 phase around Noida station as this region houses a high number of migratory laborers who resumed their work after relaxation of economic activity restrictions. The possible sources of SO_2_ emissions could be local industries, coal-based power plants, and domestic level burning activities. Stubble burning in this region could also have contributed SO_2_.^28^ Singh and Sidhu reported that about 25–30% nitrogen and phosphorus, 35–40% sulfur, and 70–75% of potassium uptake is retained in wheat residue.^32^ The concentration of SO_2_ showed a declining trend at all stations due to lockdown compared to the same time period during the previous year (*Supplemental Material Figure 2*). Overall, SO_2_ concentrations were not affected except at a few stations where they declined during the LD period.

Pollutant concentrations in the ambient atmosphere are governed by prevailing meteorological variables such as wind velocity, temperature, relative humidity, and the planetary boundary layer (PBL). Generally, high wind speed and low relative humidity help in dispersal of air pollutants compared to stagnant atmosphere.^5^,^33^ Increases in PBL height lead to larger volume of ambient atmosphere for dispersion of pollutants and are considered to be an indication of open and unstable atmosphere.^5^,^33^ Therefore, the slight decrease in pollutant concentrations during lockdown may not necessarily be due to or indicate a decrease in pollutant emissions. Since the PBL increased by more than 100%, pollutant concentrations should have been reduced by half during lockdown period under similar emission rates of pollutants during lockdown. Contrary to this theory, pollutant concentrations were not lowered by half under increased PBL height during lockdown. In addition, an increase in wind speed at some of the stations could have contributed particles in ambient atmosphere via re-suspension of surface dust. This suggests that pollutant concentration in ambient atmosphere were not lowered only due to reduced emissions of pollutants during the lockdown period, but metrological variables also played a significant role in bringing down pollution levels.^34^ Thus, it can be concluded that both lockdown and the prevailing meteorological conditions like increase in PBL and high wind speed were responsible for lower pollution levels.

Since the majority of pollution sources were not operational during lockdown, only two components, PC1 and PC2, were extracted across all monitoring stations except for Karnal and Noida where only one component was extracted. On a cumulative basis, PC1 and PC2 could explain nearly 80% of the data variance, the remaining could be associated with other pollutants not considered in the present study. The PCA analysis indicated a complex situation. PM_2.5_ and PM_10_ remained associated with one component, which is attributed to transported dust or re-suspended dust, whereas additions of NO_2_ and SO_2_ in PC1 were site specific. This could be linked to vehicular movement or diffused coal burning which were not uniform during lockdown. Some stations experienced more traffic movement due to their proximity to essential service stores or greater population. Similarly, there was more diffused coal burning for domestic purposes around stations with a primarily poor and migrant population. The current data did not allow examination of the association of O_3_ with PC2 or SO_2_ at selected stations. The formation of O_3_ requires atomic oxygen which comes from photo-dissociation of NO_2_. The possibility that chemical transformation of SO_2_ could yield atomic oxygen has yet to be explored and established and it was possible that there was an unknown source of atomic oxygen. Such possibilities are further strengthened by the negative loadings of O_3_ in PC2 at the Gurugram, Bahadurgarh and Noida sites, which shares a border with New Delhi and at IGIA-T3 in New Delhi.

## Conclusions

Air quality improved significantly in and around New Delhi after the implementation of lockdown compared to before lockdown. However, improvements in air quality due to lockdown were monitoring station and pollutant-specific. Pollutant concentrations started increasing slowly, particularly after LD2, due to relaxation of agricultural and industrial activity restrictions. At stations along national highways, air quality was initially better due to the combined effect of industrial shut down and limited vehicular movement but started to worsen on account of relaxation of restrictions and restarting of activities. The NO_2_ and O_3_ maximum concentrations varied across all sites during the entire study period, although NO_2_ is a strong precursor for O_3_ formation. Principal component analysis extracted two principal components (PC1 and PC2) and explained up to 80% cumulative variance in the data. Among all pollutants, PM_2.5_, PM_10_, and NO_2_ were associated with PC1, whereas PC2 had loadings of either O_3_ only or both O_3_ and SO_2_. Meteorological parameters, particularly wind speed, influenced PM_10_ concentrations through transport of dust and re-suspension of surface dust.

The decrease in pollution levels could not be explained entirely based on restrictions on emission sources during lockdown. Increased wind speed and PBL were also possible reasons for dispersal of pollutants and lower concentrations in ambient atmosphere. Decreased pollution load due to lockdown may lead to improvements in human health, but caution should be taken with implementation of such lockdowns in the future, as they have negative impacts on the economic and social environment of the country.
