# Supplementary material for: Air Quality Before and After COVID-19 Lockdown Phases Around New Delhi, India
Source: J Health Pollut. 2021 May 28;11(30):210602. doi: 10.5696/2156-9614-11.30.210602 (PMC8276728; doi:10.5696/2156-9614-11.30.210602)
Supplement: Supplementary file 1 [file Yadav_Supplemental_Material.docx]

**Supplemental Material**

**Supplemental Table 1;** Average Concentrations (µg m^-3^) and Percent (%) Change in Pollutant Concentrations (PM_2.5_, PM_10_, NO_2_, SO_2_ and O_3_) before and During Lockdown for 13 Monitoring Stations Around New Delhi

| **Pollutant** | **PM_2.5_**(µg m^-3^) | **PM_10_**  (µg m^-3^) | **SO_2_**  (µg m^-3^) | **NO_2_**  (µg m^-3^) | **O_3_**  (µg m^-3^) |
| --- | --- | --- | --- | --- | --- |
| 1. **ANAND VIHAR** | | | | | |
| **NAAQS** | 60 | 100 | 80 | 80 | 100 |
| **WHO** | 25 | 50 | 20 | 40 | 100 |
| **BLD** | 78.1 | 193.8 | 10.6 | 54.5 | 32.8 |
| **Mean ± SD** | 78.1±33.1 | 193.8±74 | 10.6±5.3 | 54.5±13.8 | 32.8±13.3 |
| **Range** | 19.6-176.5 | 42.5-325.8 | 3.7-29.5 | 33.3-79.5 | 16.5-70.6 |
| **LD1** | 33.09  -57.6 | 78.92  -59.2 | 9.64  -9.05 | 18.73  -65.6 | 59.63  +81.7 |
| **Mean ± SD** | 33.09±6.70 | 78.92±35.06 | 9.64±1.80 | 18.73±3.67 | 59.63±14.80 |
| **Range** | 22.69-44.99 | 39.44-191 | 7.53-12.89 | 14.68-25.41 | 35.50-81.23 |
| **LD2** | 50.6  +52.9 | 109.2  +38.3 | 15.8  +63.9 | 20.6  +9.9 | 76.3  +27.9 |
| **Mean ± SD** | 50.6±12.5 | 109.2±21.7 | 15.8±5.3 | 20.6±8.3 | 76.3±15.5 |
| **Range** | 28.6-68.7 | 79.6-140.1 | 10.1-33.8 | 10.5-27.8 | 51.5-102.5 |
| **LD3** | 82  +62.0 | 144.4  +32.2 | 22.7  +43.6 | 23.9  +16.0 | 90.6  +18.7 |
| **Mean ± SD** | 82±10.9 | 144.4±15 | 22.7±5 | 23.9±3.3 | 90.6±16.3 |
| **Range** | 70.6-96.6 | 130.7-165.2 | 20.2-28.3 | 16.5-28.7 | 68.2-106.4 |
| **LD4** | 71.94  -12.6 | 112.35  -28.5 | 14.38  -36.6 | 36.19  +51.4 | 99.9  +10.2 |
| **Mean ± SD** | 71.94±30.5 | 112.35±46.5 | 14.38±26.3 | 36.19±4.4 | 99.9±27.9 |
| **Range** | 28.5-128.9 | 35.7-177.3 | 8.09-23.6 | 12.43-115.2 | 30.4-145.4 |
| **B) NARELA** | | | | | |
| **BLD** | 71.9 | 165.9 | 17.3 | 39.6 | 47.8 |
| **Mean ± SD** | 71.9±24.6 | 165.9±61.9 | 17.3±4.6 | 39.6±8.9 | 47.8±9 |
| **Range** | 23-131.7 | 46.2-300.2 | 10.9-29.6 | 21.7-58.6 | 30.4-66.8 |
| **LD1** | 42.52  -40.8 | 106.8  -35.6 | 9.04  -47.7 | 28.81  -27.0 | 46.5  -2.71 |
| **Mean ± SD** | 42.52±16.5 | 106.8±32.7 | 9.04±3.1 | 28.81±38.1 | 46.5±17.6 |
| **Range** | 25.5-72.7 | 54.0-164.6 | 5.48-16.2 | 12.1-129.5 | 32.1-89.1 |
| **LD2** | 60.2  +41.5 | 156.5  +46.5 | 14  +54.8 | 32.6  +13.1 | 75.5  +62.3 |
| **Mean ± SD** | 60.2±19 | 156.5±38.8 | 14±4.2 | 32.6±6.5 | 75.5±11.1 |
| **Range** | 36-103.3 | 83.4-228.4 | 7.1-20.6 | 26-49.5 | 50.7-87 |
| **LD3** | 73.4  +21.9 | 177.1  +13.1 | 16.6  +18.5 | 27.2  -16.5 | 87.5  +15.8 |
| **Mean ± SD** | 73.4±21.6 | 177.1±49.5 | 16.6±5.3 | 27.2±2.8 | 87.5±12 |
| **Range** | 36.1-113.2 | 91.2-254.1 | 10.3-30.9 | 23.1-31.9 | 71.1-106.7 |
| **LD4** | 75.9  +3.4 | 219.4  +23.8 | 21.1  +27.1 | 35.7  +31.25 | 68.4  -21.8 |
| **Mean ± SD** | 75.9±35.9 | 219.4±90.0 | 21.1±10.5 | 35.7±8.4 | 68.4±17.8 |
| **Range** | 23.0-136.0 | 47.4-327.5 | 5.8-38.1 | 26.2-50.1 | 39.5-91 |
| **C) IGIA-T3** | | | | | |
| **BLD** | 55.3 | 136.3 |  | 48.2 | 12.5 |
| **Mean ± SD** | 55.3±17.7 | 136.3±46.9 |  | 48.2±34.1 | 12.5±3 |
| **Range** | 16.8-91.9 | 45-220.1 |  | 12.2-150.5 | 6.9-18.2 |
| **LD1** | 34.6  -37.4 | 72.6  -1.37 |  | 7.9  -83.6 | 22.4  +79.2 |
| **Mean ± SD** | 34.6±13.8 | 72.6±28.0 |  | 7.9±8.9 | 22.4±5.4 |
| **Range** | 16.6-64.4 | 29.0-135.2 |  | -20.1-18.5 | 11.5-28.7 |
| **LD2** | 38.7  +11.8 | 91.3  +25.7 |  | 15.2  +92.4 | 27.7  +23.6 |
| **Mean ± SD** | 38.7±12.7 | 91.3±32.4 |  | 15.2±11.4 | 27.7±4.8 |
| **Range** | 20.5-56.5 | 40.5-168.9 |  | 3.5-38.6 | 17.5-34.9 |
| **LD3** | 49.6  +28.1 | 131.1  +43.5 |  | 5.5  -63.8 | 34.6  +24.9 |
| **Mean ± SD** | 49.6±14.3 | 131.1±40.5 |  | 5.5±2.4 | 34.6±4.7 |
| **Range** | 30.6-90.3 | 84.4-212.2 |  | 1.9-10.2 | 28.7-45 |
| **LD4** | 53.5  +7.8 | 140  +6.7 |  | 5.3  -3.6 | 26.5  -23.4 |
| **Mean ± SD** | 53.5±20.6 | 140±51.8 |  | 5.3±4.3 | 26.5±4.7 |
| **Range** | 23.4-100.2 | 43.8-219.7 |  | 1.04-12.4 | 15.9-33.7 |
| **D) NAJAFGARH** | | | | | |
| **BLD** | 69.3 | 133.7 | 13.8 | 26.5 | 46.7 |
| **Mean ± SD** | 69.3±27.4 | 133.7±49.1 | 13.8±4.1 | 26.5±6.7 | 46.7±13.1 |
| **Range** | 15.5-114.3 | 25.2-219.6 | 6.5-21.9 | 15.3-43.8 | 18.5-80 |
| **LD1** | 50.6  -26.9 | 147.1  +10.0 | 15.0  +8.6 | 11.3  -57.3 | 49.1  +5.1 |
| **Mean ± SD** | 50.6±17.3 | 147.1±41.4 | 15.0±4.0 | 11.3±4.4 | 49.1±13.3 |
| **Range** | 22.4-78.6 | 98.7-225.1 | 9.23-22.0 | 4.58-19.42 | 26.6-66.9 |
| **LD2** | 42  -16.9 | 135  -8.2 | 10.6  -29.3 | 13.5  +19.4 | 28  -42.9 |
| **Mean ± SD** | 42±18.1 | 135±48.7 | 10.6±2.8 | 13.5±5.5 | 28±12.6 |
| **Range** | 14.6-78.2 | 40.6-209.9 | 6.6-19.2 | 7.5-25.9 | 2.2-54.9 |
| **LD3** | 54.8  +30.4 | 129.2  -4.29 | 12.2  +15.0 | 12.2  -10.6 | 23.8  -15 |
| **Mean ± SD** | 54.8±24 | 129.2±40.8 | 12.2±1.8 | 12.2±1.9 | 23.8±5.1 |
| **Range** | 21.6-109.9 | 53-202.7 | 8.6-14.7 | 9.9-15.4 | 15.6-31 |
| **LD4** | 58.3  +6.3 | 175.8  +36.0 | 13.2  +8.1 | 16.4  +34.4 | 9.6  -59.6 |
| **Mean ± SD** | 58.3±36.6 | 175.8±79.2 | 13.2±4.4 | 16.4±5.4 | 9.6±2.5 |
| **Range** | 16.2-133.4 | 35.3-278.4 | 7.64-22.9 | 6.99-23.2 | 5.5-13.3 |
| **E) OKHLA** | | | | | |
| **BLD** | 61.5 | 146.8 | 15.9 | 36.3 | 29.8 |
| **Mean ± SD** | 61.5±19.5 | 146.8±50.2 | 15.9±4.2 | 36.3±10.3 | 29.8±9.1 |
| **Range** | 19.2-100.3 | 41.4-235.7 | 8.2-26.2 | 21.4-54.7 | 4.7-47.1 |
| **LD1** | 38.0  -38.2 | 79.4  -45.9 | 17.4  +9.4 | 13.4  -63.0 | 43.7  +46.6 |
| **Mean ± SD** | 38±14.8 | 79.4±30.2 | 17.4±3.20 | 13.4±4.6 | 43.7±9.3 |
| **Range** | 19.0-78.6 | 32.4-153.8 | 12.9-25.5 | 7.5-25.3 | 24.3-59.1 |
| **LD2** | 43.2  +13.6 | 107.9  +35.8 | 14.4  -17.2 | 15.3  +14.1 | 45.4  +3.8 |
| **Mean ± SD** | 43.2±14.5 | 107.9±36.2 | 14.4±3.6 | 15.3±5.4 | 45.4±8.2 |
| **Range** | 23.6-70.9 | 47.2-197 | 9.3-22.4 | 7.1-22.4 | 29.9-56.2 |
| **LD3** | 46.4  +7.4 | 114.4  +5.7 | 13.5  -6.25 | 17.7  +15.6 | DATA NOT AVAILABLE |
| **Mean ± SD** | 46.4±13.8 | 114.4±29.8 | 13.5±2.5 | 17.7±5.7 |  |
| **Range** | 23.3-78.9 | 55.8-165 | 10.8-21.2 | 10.9-31.9 |  |
| **LD4** | 47.5  +2.37 | 153.3  +34 | 16.5  +22.2 | 33.8  +90.6 | 13.9 |
| **Mean ± SD** | 47.5±24.3 | 153.3±62.5 | 16.5±4.5 | 33.8±11.6 | 13.9±14.1 |
| **Range** | 19.4-102.6 | 38.2-258.0 | 10.7-24.0 | 15.4-49.3 | 1.9-31.7 |
| **F) JAHANGIRPURI** | | | | | |
| **BLD** | 104.5 | 224.3 | 22.8 | 50.6 | 37.7 |
| **Mean ± SD** | 104.5±21.4 | 224.3±46.6 | 22.8±4.7 | 50.6±9.9 | 37.7±13.6 |
| **Range** | 61.4-140.8 | 125.9-317.2 | 15.9-34.6 | 33.2- 65.7 | 10.5-64.8 |
| **LD1** | 122.29  + 17.02 | 271.04  + 20.52 | 30.15  + 32.23 | 52.65  + 4.05 | 53.67  + 42.3 |
| **Mean ± SD** | 122.2±35.74 | 271±54.62 | 30.1±6.94 | 52.6±8.91 | 53.6±9.11 |
| **Range** | 72.17-240.08 | 182.50-400.09 | 21.75-46.35 | 36.47-67.35 | 36.31-78.95 |
| **LD2** | 96.6  - 21 | 262.4  - 3.18 | 26.5  - 12.10 | 50.6  - 3.8 | 63.4  + 18.1 |
| **Mean ± SD** | 96.6±25.7 | 262.4±84.1 | 26.5±6.5 | 50.6±10.2 | 63.4±11.7 |
| **Range** | 33.9-125.9 | 76.9-378.3 | 15.5-36.6 | 35.2-68.1 | 41.5-84.6 |
| **LD3** | 114.5  + 18.5 | 337.9  + 28.7 | 28.6  + 7.92 | 49.8  - 1.58 | 63.6  + 0.3 |
| **Mean ± SD** | 114.5±40.1 | 337.9±93.4 | 28.6±7.3 | 49.8±8.2 | 63.6±11.7 |
| **Range** | 52.5-199.3 | 151.2-471.7 | 17.3-45.2 | 36.9-63.1 | 43.3-84.2 |
| **LD4** | 83.64  - 26.9 | 235.58  - 30.2 | 27.32  - 4.47 | 55.79  + 12.02 | 64.17  + 0.89 |
| **Mean ± SD** | 83.6±21.52 | 235.5±67.20 | 27.3±5.94 | 55.7±16.81 | 64.1±12.27 |
| **Range** | 53.58-129.02 | 126.38-357.89 | 15.78-35.75 | 34.55-81.67 | 30.96-79.38 |
| **G) SONIPAT** | | | | | |
| **BLD** | 33.2 | 132.9 | 27.6 | 46.8 | 20.2 |
| **Mean ± SD** | 33.2±13.1 | 132.9±43.3 | 27.6±1.6 | 46.8±11.5 | 20.2±9.1 |
| **Range** | 12.7-71.2 | 69.7-212.7 | 23.9-33.1 | 35.4-75.3 | 7.7-35.0 |
| **LD1** | 22.64  - 31.80 | 84.89  - 36.1 | 28.00  + 1.44 | 55.79  + 19.20 | 14.55  - 27.9 |
| **Mean ± SD** | 22.6±7.17 | 84.8±26.60 | 28±0.43 | 55.7±10.62 | 14.5±4.39 |
| **Range** | 10.83-39.13 | 34.02-148.49 | 27.05-28.79 | 38.85-74.31 | 10.44-24.59 |
| **LD2** | 34.9  + 54.1 | 149.3  + 75.8 | 27.9  - 0.35 | 50.7  + 9.12 | 16.2  + 11.34 |
| **Mean ± SD** | 34.9±12.3 | 149.3±45.6 | 27.9±0.5 | 50.7±20.4 | 16.2±3.9 |
| **Range** | 19.1-63.8 | 67.4-237.3 | 27.0-28.7 | 27.1-96.5 | 11.3-24.6 |
| **LD3** | 38.3  - 9.74 | 176.9  + 18.48 | 27.0  - 3.22 | 57.6  + 1.36 | 18.1  + 11.7 |
| **Mean ± SD** | 38.3±14.3 | 176.9±65.3 | 27±1.5 | 57.6±9.6 | 18.1±2.9 |
| **Range** | 15.6-68.9 | 51.0-289.2 | 22.3-28.4 | 39.1-78.5 | 14.4-25.2 |
| **LD 4** | 40.54  + 5.84 | 167.65  - 5.22 | 27.85  + 3.14 | 43.11  - 25.8 | 26.34  + 45.5 |
| **Mean ± SD** | 40.5±16.23 | 167.6±55.38 | 27.8±0.69 | 43.1±12.18 | 26.3±3.87 |
| **Range** | 13.6-69.17 | 73.1-242.26 | 26.66-29.5 | 27-69.16 | 22.09-37.02 |
| **H) PANIPAT** | | | | | |
| **BLD** | 38.7 | 94.8 | 20.2 | 47.6 | 27.9 |
| **Mean ± SD** | 38.7±9.9 | 94.8±35.4 | 20.2±4.5 | 47.6±10.4 | 27.9±9.3 |
| **Range** | 21.4-60.9 | 45.9-162.3 | 12.7-30.1 | 27.3-70.4 | 14.3-45.5 |
| **LD1** | 38.81  + 0.28 | 153.84  + 62.2 | 18.52  - 8.31 | 44.75  - 5.98 | 34.98  + 25.3 |
| **Mean ± SD** | 38.8±9.70 | 153.8±90.48 | 18.5±5.79 | 44.7±13.86 | 34.9±8.00 |
| **Range** | 24.54-59.44 | 78.42-487.59 | 13.75-64.46 | 5.82-28.98 | 18.97-48.48 |
| **LD2** | 42.7  + 10.0 | 177.2  + 15.18 | 18.4  - 0.64 | 33.7  - 24.6 | 50.7  - 44.9 |
| **Mean ± SD** | 42.7±15.5 | 177.2±85.3 | 18.4±8.0 | 33.7±15.0 | 50.7±18.2 |
| **Range** | 16.0-71.7 | 29.5-309.2 | 6.3-31.2 | 12.3-66.1 | 21.1-83.0 |
| **LD3** | 82.5  + 9.11 | 276.4  + 55.9 | 47.5  + 158.1 | 23.8  - 29.3 | 34.1  - 32.7 |
| **Mean ± SD** | 82.5±26.9 | 276.4±71.5 | 47.5±5.3 | 23.8±14.9 | 34.1±11.2 |
| **Range** | 39.7-131.9 | 151.7-388.5 | 11.5-29.3 | 25.3-78.9 | 17.4-48.8 |
| **LD4** | 48.06  - 71.66 | 211.23  - 30.85 | 18.74  - 60.54 | 23.24  - 2.40 | 22.17  - 34.9 |
| **Mean ± SD** | 48.0±16.41 | 211.2±93.62 | 18.7±5.18 | 23.2±15.02 | 22.1±3.68 |
| **Range** | 25.69-79.99 | 95.42-395.58 | 8.52-25.36 | 6.47-45.99 | 16.35-28.36 |
| 1. **KARNAL** | | | | | |
| **BLD** | 51.2 | 100.5 | 42.2 | 17.4 | 18.9 |
| **Mean ± SD** | 51.2±19.8 | 100.5±44.6 | 42.2±22.1 | 17.4±4.7 | 18.9±3.7 |
| **Range** | 23.3-105.1 | 39.5-198.7 | 13.3-86.2 | 10.4-26.4 | 11.6-27.0 |
| **LD1** | 32.25  - 36.5 | 64.9  - 35.4 | 28.1  - 33.4 | 6.1  - 64.9 | 25.5  + 34.9 |
| **Mean ± SD** | 32.2±9.8 | 64.9±31.4 | 28.1±26.8 | 6.1±2.4 | 25.5±3.2 |
| **Range** | 17-55.6 | 21.2-139.39 | 2.29-88.3 | 2.24-11.5 | 20.1-33.5 |
| **LD2** | 51.9  + 60.93 | 82.7  + 27.42 | 39.3  + 39.8 | 11.1  + 81.9 | 38.9  + 52.5 |
| **Mean ± SD** | 51.9±19.8 | 82.7±38.7 | 39.3±27.6 | 11.1±3.4 | 38.9±11.7 |
| **Range** | 23.6-78 | 36.8-195.2 | 3.04-84.13 | 5.48-16.1 | 24.7-63.3 |
| **LD3** | 51.5  - 0.77 | 79.5  - 3.86 | 29.9  - 23.9 | 11.5  + 3.6 | 45.5  + 16.9 |
| **Mean ± SD** | 51.5±21.4 | 79.5±27.7 | 29.9±25.8 | 11.5±3.29 | 45.5±12.1 |
| **Range** | 24-88.4 | 38.3-122.3 | 71.8-77.8 | 7.13-18.26 | 26.7-62.3 |
| **LD4** | 59.8  + 16.1 | 106  + 33.3 | 44.7  + 49.4 | 14.3  + 24.3 | 38  -16.4 |
| **Mean ± SD** | 59.8±25.4 | 106±46.2 | 44.7±29.5 | 14.3±4.4 | 38±9.07 |
| **Range** | 21.8-96.9 | 33.2-189.1 | 1.49-87.7 | 7.6-23.7 | 19.4-56.4 |
| **J) BAHADURGARH** | | | | | |
| **BLD** | 61.6 | 101.2 | 17.6 | 23.6 | 34.5 |
| **Mean ± SD** | 61.6±20.8 | 101.2±37.3 | 17.6±9.1 | 23.6±12.1 | 34.5±13.1 |
| **Range** | 20.4-100.8 | 26.9-163.9 | 2.7-41.3 | 10.6-61.0 | 18.4-79.8 |
| **LD1** | 36.7  - 40.4 | 66.6  - 34.1 | 12.6  - 28.4 | 16.8  - 28.8 | 41.9  + 21.4 |
| **Mean ± SD** | 36.7±15.3 | 66.6±29.4 | 12.6±5.5 | 16.8±3.3 | 41.9±4.9 |
| **Range** | 12.5-72.2 | 18.8-143.9 | 3.59-22.9 | 7.3-22.4 | 34.6-52.2 |
| **LD2** | 47.1  + 28.3 | 99.2  + 48.9 | 10.3  - 18.2 | 20.2  + 20.2 | 61.3  + 46.3 |
| **Mean ± SD** | 47.1±15.3 | 99.2±31.5 | 10.3±5 | 20.2±5.2 | 61.3±19.4 |
| **Range** | 20.4-69.9 | 37.8-162.8 | 4.3-21.5 | 13.2-30.7 | 34.6-94.8 |
| **LD3** | 49.5  + 5.09 | 109.2  + 10.08 | 8.27  - 19.7 | 27.1  + 34.1 | 39.8  - 35.07 |
| **Mean ± SD** | 49.5±22.2 | 109.2±58.2 | 8.27±5.11 | 27.1±23.2 | 39.8±18.9 |
| **Range** | 22.9-75.4 | 36.4-196.2 | 1.73-16.18 | 1.74-78.0 | 6.42-53.4 |
| **LD4** | 45.8  - 7.47 | 112.2  + 2.74 | 6.18  - 25.2 | 22.8  - 15.8 | 51.5  + 29.3 |
| **Mean ± SD** | 45.8±37 | 112.2±75.6 | 6.18±5.0 | 22.8±13.0 | 51.5±29.1 |
| **Range** | 14.9-134.3 | 31.27-220.4 | 3.17-19.4 | 10.8-34.4 | 27.9-88.6 |
| **K) GURUGRAM** | | | | | |
| **BLD** | 71.3 | 154.5 | 19.1 | 24.7 | 59.0 |
| **Mean ± SD** | 71.3±26.0 | 154.5±56.7 | 19.1±2.3 | 24.7±2.7 | 59±9.9 |
| **Range** | 22.4-120.4 | 38.9-249.7 | 15.5-23.8 | 21.2- 30.3 | 42.2-75.7 |
| **LD1** | 38.4  - 46.1 | 84.2  - 45.5 | 14.6  - 23.5 | 14.9  - 39.6 | 70.1  + 18.8 |
| **Mean ± SD** | 38.4±15 | 84.2±32.2 | 14.6±1.11 | 14.9±1.96 | 70.1±12 |
| **Range** | 18.1-68.2 | 43.3-177.3 | 13.07-17.5 | 12.11-19.4 | 45.4-88.3 |
| **LD2** | 48.4  + 26 | 116.8  + 38.7 | 10.6  - 27.3 | 14  -6.0 | 86  + 22.6 |
| **Mean ± SD** | 48.4±16.2 | 116.8±38.7 | 10.6±1.1 | 14±2.1 | 86±15.7 |
| **Range** | 25.7-67.6 | 62.2-210.7 | 7.9-12.6 | 9.7-17.6 | 50.8-113.0 |
| **LD3** | 48.5  + 0.20 | 114.5  - 1.96 | 9.5  - 10.3 | 12.5  - 10.7 | 106.4  + 23.7 |
| **Mean ± SD** | 48.5±11.9 | 114.5±31.7 | 9.5±3.9 | 12.5±1.6 | 106.4±14.2 |
| **Range** | 22.1-69.1 | 51.4-190.2 | 7.5-23.1 | 10.7-17.5 | 81.5-137.2 |
| **LD4** | 69.7  + 43.7 | 188.2  + 64.3 | 9.5  NA | 16.7  + 33.6 | 86.1  - 19.0 |
| **Mean ± SD** | 69.7±34.3 | 188.2±87.9 | 9.5±7.3 | 16.7±2.5 | 86.1±13.4 |
| **Range** | 29.4-142.1 | 73.5-341.1 | 4.9-34.8 | 13.1-22.1 | 50.9-106.4 |
| **L) FARIDABAD** | | | | | |
| **BLD** | 71.9 | 174.5 | 6.5 | 7.9 | 26.6 |
| **Mean ± SD** | 71.9±19.9 | 174.5±52.7 | 6.5±1.3 | 7.9±3.4 | 26.6±5.3 |
| **Range** | 26.7-110.8 | 46.9-288.9 | 3.9-9.7 | 4.1-18.2 | 18.6-36.5 |
| **LD1** | 31.47  - 55.6 | 85  - 51.2 | 5.75  - 11.5 | 9.9  + 25.3 | 23.2  - 12.78 |
| **Mean ± SD** | 31.47±21 | 85±35.7 | 5.75±1.35 | 9.9±3.17 | 23.2±7.8 |
| **Range** | 7.11-77.2 | 31.25-157.1 | 4.89-6.87 | 4.23-11.1 | 9.65-30.18 |
| **LD2** | 27.2  - 13.5 | 139.0  + 63.5 | 5.4  - 6.08 | 11.0  + 11.1 | 19.8  - 14.6 |
| **Mean ± SD** | 27.2±29.4 | 139.0±57.7 | 5.4±1.3 | 11.0±0.01 | 19.8±6.8 |
| **Range** | 9.42-119.5 | 64.5-235.1 | 3.94-10.3 | 10.97-11.06 | 8.75-30.8 |
| **LD3** | 49.9  + 83.45 | 142.5  + 2.51 | 2.97  - 45 | 11.0  NA | 17.04  - 13.93 |
| **Mean ± SD** | 49.9±48.7 | 142.5±41.77 | 2.97±1.13 | 11.0±0.01 | 17.04±19.1 |
| **Range** | 11.6-146.0 | 64.7-237.6 | 1.81-6.49 | 10.9-11.04 | 2.22-49.4 |
| **LD4** | 85.8  + 71.9 | 175.5  + 23.15 | 2.38  - 19.8 | 11.0  NA | 37.6  + 120.6 |
| **Mean ± SD** | 85.8±42.8 | 175.5±61.5 | 2.38±1.23 | 11.0±0.01 | 37.6±8.39 |
| **Range** | 9.41-144.2 | 70.6-292.7 | 1.37-5.26 | 10.9-11 | 20.6-47.2 |
| **M) NOIDA** | | | | | |
| **BLD** | 57.4 | 136.7 | 12.7 | 38.5 | 29.3 |
| **Mean ± SD** | 57.4±21.6 | 136.7±51.2 | 12.7±4.0 | 38.5±14.0 | 29.3±5.8 |
| **Range** | 17.8-110.0 | 37.0-240.3 | 4.4-20.3 | 12.9-72.2 | 18.5-39.0 |
| **LD1** | 33.6  - 41.4 | 86.8  - 36.5 | 12.1  - 4.72 | 12.0  - 208.66 | 18.3  - 37.54 |
| **Mean ± SD** | 33.6±13.1 | 86.8±35.4 | 12.1±4.8 | 12.0±2.68 | 18.3±3.8 |
| **Range** | 16.8-57 | 31.72-186.8 | 6.25-26.4 | 8.9-18.4 | 12.18-25.0 |
| **LD2** | 49  + 45.8 | 140.4  + 61.75 | 12.8  + 5.7 | 13.4  + 11.6 | 15.9  - 13.11 |
| **Mean ± SD** | 49±30.9 | 140.4±87.2 | 12.8±7.7 | 13.4±7.5 | 15.9±8.8 |
| **Range** | 17.3-103.1 | 53.2-288.09 | 6.32-28.05 | 8.3-23.6 | 9.96-27.7 |
| **LD3** | 45.6  - 6.9 | 120  - 14.5 | 14.0  + 9.3 | 14.3  + 6.7 | 2.05  - 87.4 |
| **Mean ± SD** | 45.6±18.7 | 120±47.3 | 14.0±5.0 | 14.3±6.02 | 2.05±1.7 |
| **Range** | 24.9-74.6 | 58.5-177.25 | 8.72-20.9 | 9.84-25.5 | 0.4-6.26 |
| **LD4** | 46.5  + 1.97 | 170.7  + 42.25 | 15.78  + 12.71 | 21.2  + 48.2 | 1.86  - 9.26 |
| **Mean ± SD** | 46.5±23.5 | 170.7±73.4 | 15.78±8.89 | 21.2±8.0 | 1.86±1.05 |
| **Range** | 16.8-85.6 | 33.6-269.7 | 3.28-32.7 | 9.78-32.05 | 0.8-3.23 |
| <10%  10-30%  30-50%  >50%  <10%  10-30%  30-50%  >50%  (-) Decrease in conc.  (+) Increase in conc. | | | | | |

Percentage change was calculated relative to the values in the preceding phase.

Abbreviations: BLD: Before lockdown (BLD: 1– 24March 2020); Lockdown phase 1 (LD1:25March– 14 April 2020) when there was a complete lockdown; Lockdown phase 2 (LD2:15 April– 3 May 2020) when selected agricultural and industrial activities opened; Lockdown phase 3 (LD3: 4- 17 May 2020) and Lockdown phase 4 (LD4: 18- 31 May 2020)when more industrial and construction activities were allowed; NAAQS: National Ambient Air Quality Standards; WHO: World Health Organization.

**Supplemental Table 2:** Principal Component Analysis for Pollutants (PM_2.5_, PM_10_, NO_2_, SO_2_ and O_3_) over Selected Monitoring Station Around New Delhi before and During Lockdown

|  | Anand Vihar_BLD | | Narela_BLD | | IGIA-T3_BLD | | Najafgarh_BLD | | Okhala_BLD | |
| --- | --- | --- | --- | --- | --- | --- | --- | --- | --- | --- |
|  | PC1 | PC2 | PC1 | PC2 | PC1 | PC2 | PC1 | PC2 | PC1 | PC2 |
| PM_2.5_ | **0.88** | 0.20 | **0.96** | -0.01 | **0.92** | 0.26 | **0.85** | 0.37 | **0.88** | 0.30 |
| PM_10_ | **0.98** | -0.08 | **0.96** | 0.15 | **0.90** | 0.07 | **0.89** | 0.34 | **0.96** | 0.06 |
| NO_2_ | **0.69** | -0.43 | 0.30 | **0.89** | 0.57 | -0.54 | 0.29 | 0.73 | **0.86** | -0.15 |
| SO_2_ | 0.18 | **0.89** | 0.87 | 0.10 | NA | NA | **0.94** | -0.18 | 0.57 | **0.69** |
| O_3_ | -0.24 | **0.88** | 0.63 | -0.66 | 0.26 | **0.84** | 0.02 | -0.83 | -0.13 | **0.95** |
| Eigen value | 2.40 | 1.68 | 3.09 | 1.26 | 2.09 | 1.02 | 2.81 | 1.19 | 2.92 | 1.35 |
| % variance | 48.05 | 33.61 | 61.75 | 25.10 | 52.33 | 25.54 | 56.24 | 23.84 | 58.45 | 27.01 |
| Cumulative% | 48.05 | 81.67 | 61.75 | 86.85 | 52.33 | 77.87 | 56.24 | 80.08 | 58.45 | 85.46 |
|  | Anand Vihar_LD | | Narela_LD | | IGIA-T3_LD | | Najafgarh_LD | | Okhla_LD | |
|  | PC1 | PC2 | PC1 | PC2 | PC1 | PC2 | PC1 | PC2 | PC1 | PC2 |
| PM_2.5_ | **0.96** | 0.14 | **0.95** | 0.15 | **0.93** | 0.06 | **0.92** | -0.04 | **0.87** | 0.40 |
| PM_10_ | **0.88** | 0.13 | **0.87** | 0.33 | **0.89** | 0.07 | **0.87** | -0.10 | **0.86** | -0.15 |
| NO_2_ | **0.84** | -0.32 | 0.01 | **0.93** | 0.17 | **0.90** | **0.73** | -0.41 | **0.85** | -0.26 |
| SO_2_ | **0.72** | 0.34 | **0.93** | 0.05 | NA | NA | **0.72** | 0.54 | NA | NA |
| O_3_ | 0.09 | **0.95** | 0.38 | 0.55 | 0.59 | -0.57 | -0.15 | **0.93** | -0.09 | **0.98** |
| Eigen value | 2.95 | 1.10 | 2.94 | 1.00 | 2.04 | 1.13 | 2.69 | 1.30 | 2.24 | 1.12 |
| % variance | 59.17 | 22.01 | 58.85 | 20.07 | 51.00 | 28.40 | 53.86 | 26.06 | 56.07 | 30.04 |
| Cumulative% | 59.17 | 81.18 | 58.85 | 78.90 | 51.00 | 79.41 | 53.86 | 79.93 | 56.07 | 86.11 |

|  | Jahagirpuri_BLD | | | Sonipat_BLD | | | Panipat BLD | | | | Karnal_ BLD | | | Bahadurgarh_BLD | | | |
| --- | --- | --- | --- | --- | --- | --- | --- | --- | --- | --- | --- | --- | --- | --- | --- | --- | --- |
|  | PC1 | PC2 | | PC1 | | PC2 | PC1 | | PC2 | | PC1 | | PC2 | PC1 | | PC2 | |
| PM_2.5_ | **0.94** | 0.14 | | **0.97** | | -0.11 | -0.16 | | **0.95** | | **0.85** | | 0.49 | **0.96** | | 0.13 | |
| PM_10_ | **0.91** | 0.33 | | **0.85** | | -0.15 | 0.48 | | **0.83** | | **0.91** | | 0.33 | **0.90** | | 0.17 | |
| NO_2_ | **0.88** | -0.20 | | 0.26 | | -0.43 | **0.84** | | 0.13 | | **0.83** | | -0.42 | 0.29 | | 0.73 | |
| SO_2_ | 0.47 | **0.81** | | -0.32 | | **0.74** | **0.66** | | 0.49 | | 0.03 | | **0.89** | **0.86** | | 0.03 | |
| O_3_ | -0.15 | **0.94** | | 0.44 | | **0.79** | **0.81** | | -0.08 | | 0.21 | | **0.92** | -0.06 | | **0.87** | |
| Eigen value | 2.91 | 1.50 | | 2.09 | | 1.32 | 2.56 | | 1.33 | | 2.81 | | 1.62 | 2.72 | | 1.17 | |
| % variance | 58.31 | 30.10 | | 41.87 | | 26.39 | 51.38 | | 26.66 | | 56.22 | | 32.55 | 54.50 | | 23.48 | |
| Cumulative% | 58.31 | 88.41 | | 41.87 | | 68.26 | 51.38 | | 78.05 | | 56.22 | | 88.77 | 54.50 | | 77.99 | |
|  | Jahagirpuri_LD | | | Sonipat_ LD | | | Panipat_LD | | | | Karnal_LD | |  | Bahadurgarh_LD | | | |
|  | PC1 | PC2 | | PC1 | | PC2 | PC1 | | PC2 | | PC1 | |  | PC1 | | PC2 | |
| PM_2.5_ | **0.86** | -0.27 | | **0.89** | | -0.09 | **0.92** | | 0.05 | | **0.93** | |  | **0.86** | | 0.08 | |
| PM_10_ | **0.87** | -0.03 | | **0.92** | | 0.12 | **0.91** | | -0.07 | | **0.82** | |  | **0.87** | | -0.07 | |
| NO_2_ | **0.67** | 0.26 | | -0.22 | | **0.80** | 0.23 | | **0.72** | | **0.82** | |  | 0.49 | | 0.33 | |
| SO_2_ | **0.82** | 0.14 | | -0.30 | | -0.62 | **0.69** | | 0.49 | | **0.64** | |  | 0.28 | | **0.75** | |
| O_3_ | 0.03 | **0.95** | | **0.57** | | 0.04 | -0.14 | | **0.75** | | 0.45 | |  | 0.12 | | -0.80 | |
| Eigen value | 2.60 | 1.00 | | 2.10 | | 1.10 | 2.40 | | 1.20 | | 2.80 | |  | 1.96 | | 1.19 | |
| % variance | 51.90 | 20.90 | | 42.20 | | 21.10 | 47.40 | | 23.80 | | 56.14 | |  | 39.20 | | 23.88 | |
| Cumulative% | 51.90 | 72.80 | | 42.20 | | 63.20 | 47.40 | | 71.20 | | 56.14 | |  | 39.21 | | 63.09 | |
|  | | | Gurugram_BLD | | | | | Faridabad_BLD | | | | Noida _BLD | | | | |  |
|  | | | PC1 | | PC2 | | | PC1 | | PC2 | | PC1 | | | PC2 | |  |
| PM_2.5_ | | | **0.91** | | -0.03 | | | **0.89** | | -0.03 | | **0.96** | | | 0.08 | |  |
| PM_10_ | | | **0.92** | | 0.13 | | | **0.96** | | -0.04 | | **0.97** | | | -0.10 | |  |
| NO_2_ | | | **0.82** | | 0.36 | | | 0.22 | | **0.56** | | **0.71** | | | -0.35 | |  |
| SO_2_ | | | 0.04 | | -0.80 | | | -0.29 | | **0.86** | | **0.77** | | | 0.25 | |  |
| O_3_ | | | 0.30 | | **0.73** | | | -0.09 | | **0.81** | | 0.02 | | | **0.93** | |  |
| Eigen value | | | 2.66 | | 1.07 | | | 2.04 | | 1.50 | | 2.97 | | | 1.07 | |  |
| % variance | | | 53.35 | | 21.55 | | | 40.98 | | 30.08 | | 59.49 | | | 21.41 | |  |
| Cumulative% | | | 53.35 | | 74.91 | | | 40.98 | | 71.06 | | 59.49 | | | 80.91 | |  |
|  | | | Gurugram _LD | | | | | Faridabad_LD | | | | Noida_LD | | | | |  |
|  | | | PC1 | | PC2 | | | PC1 | |  | | PC1 | | |  | |  |
| PM_2.5_ | | | **0.94** | | 0.08 | | | **0.86** | |  | | **0.90** | | |  | |  |
| PM_10_ | | | **0.94** | | 0.14 | | | **0.84** | |  | | **0.88** | | |  | |  |
| NO_2_ | | | 0.39 | | **0.82** | | | 0.42 | |  | | **0.87** | | |  | |  |
| SO_2_ | | | -0.61 | | 0.42 | | | -0.62 | |  | | **0.87** | | |  | |  |
| O_3_ | | | 0.18 | | -0.90 | | | **0.62** | |  | | -0.46 | | |  | |  |
| Eigen value | | | 2.33 | | 1.65 | | | 2.38 | |  | | 3.31 | | |  | |  |
| % variance | | | 46.69 | | 33.02 | | | 47.78 | |  | | 66.29 | | |  | |  |
| Cumulative% | | | 46.69 | | 79.71 | | | 47.78 | |  | | 66.29 | | |  | |  |

Abbreviations: BLD: Before lockdown (BLD: 1– 24March 2020); Lockdown phase 1 (LD1: 25March– 14 April 2020); Lockdown phase 2 (LD2: 15 April– 3 May 2020); Lockdown phase 3 (LD3: 4- 17 May 2020) and Lockdown phase 4 (LD4: 18- 31 May 2020)

**Supplemental Figure 1:** Average mass concentrations of PM_2.5_, PM_10_, NO_2_, SO_2_ and O_3_ at 13 locations in and around New Delhi before lockdown (BLD: 1– 24 March 2020), Lockdown phase 1 (LD1: 25 March– 14 April 2020), Lockdown phase 2 (LD2: 15 April– 3 May 2020), Lockdown phase 3 (LD3: 4 – 17 May 2020) and Lockdown phase 4 (LD4: 18 – 31 May 2020).

**Supplemental Figure 2:** Comparison of average mass concentrations of PM_2.5_, PM_10_, NO_2_, SO_2_ and O_3_ during before lockdown (BLD: 1– 24March 2020), Lockdown phase 1 (LD1: 25March– 14 April 2020), Lockdown phase 2 (LD2: 15 April– 3 May 2020), Lockdown phase 3 (LD3: 4- 17 May 2020) and Lockdown phase 4 (LD4: 18– 31 May 2020) for the same time periods during the year 2019 at selected monitoring stations
